# Supplementary material for: Alternating quarantine for sustainable epidemic mitigation
Source: Nat Commun. 2021 Jan 11;12:220. doi: 10.1038/s41467-020-20324-8 (PMC7801583; doi:10.1038/s41467-020-20324-8)
Supplement: Supplementary file 1 — Supplementary Information [file 41467_2020_20324_MOESM1_ESM.pdf]

Alternating quarantine for sustainable epidemic mitigation

Supplementary Information

Contents

1 Modeling the spread **2**

1.1 The unmitigated spread 2

1.1.1 Population network 2

1.1.2 Temporality 2

1.2 Mitigation 3

1.3 Disease dynamics 4

1.4 Evaluating α and β 6

1.5 Evaluating R_0 7

2 Results obtained under a scale-free A_{ij} **9**

3 Synergistic measures **11**

3.1 Alternating quarantine under selective isolation 11

3.2 Alternating quarantine with population-wide testing 11

4 Data analysis and parameter selection **15**

4.1 Constructing the distributions $P_{\mathbb{X}}(t)$ 15

4.2 Estimating the infection growth β 15

4.3 Estimating the household size distribution $P(m)$ 16

Supplementary Note 1

Modeling the spread

1.1 The unmitigated spread

1.1.1 Population network

We consider M households $h = 1, \ldots, M$, each including m_h individuals, where m_h is a random variable extracted from the household size distribution $P(m)$ (Supplementary Table 5). Together these households comprise the total population, in which the number of individuals $i = 1, \ldots, N$ is given by $N = \sum_{h=1}^{M} m_h$. Hence, the i-th individual, resides together with her $m_h - 1$ cohabitants at her household $h(i)$. Taking $M = 4 \times 10^3$, and the average household size to be $\langle m \rangle = 2.5$, we arrive at a total population of $N = 10^4$ individuals.

To construct the social network G_{ij}, we consider two types of links: Within a household there are no barriers, hence the in-house connection network B_{ij} is simply a union of disjoint cliques representing $(i, j) \in B_{ij} \iff h(i) = h(j)$. This results in M isolated cliques whose size is distributed via $P(m)$. Out of home connections, A_{ij}, can be potentially drawn between any pair of nodes i, j, capturing external social links, occurring at work, school or other public places. The external network A_{ij} can be admit any desired degree distribution $P(k)$ via the configuration model framework [1]. In our simulations we used two archetypal constructions - the Erdős-Rényi (ER) random graph (main text), in which $P(k)$ is bounded, and a scale-free (SF) network, where $P(k) \sim k^{-3}$ (Sec. 2). In both cases we set the average degree to $\langle k \rangle = 15$. The final network G_{ij} contains all links in A_{ij} and B_{ij}.

1.1.2 Temporality

The links in G_{ij} are not constantly active. Rather, they represent *potential* infectious interactions, switching between periods of activity, when infections can take place, and inactivity, when infections are barred. Throughout the daily cycle we have A_{ij} active during the day, 8:00 AM to 8:00 PM, and B_{ij} active during the after-hours, 8:00 PM to 8:00 AM the next day. This captures a typical routine, in which individuals interact sporadically, *i.e.* links are switched on and off, out of home in the day-time, and in-house at night.

Dividing each day into 15 minute segments, Δt, we generate a random sequence of temporal activity/inactivity instances for A_{ij} and B_{ij}. During the day, the probability for activation of each link in A_{ij} per interval Δt is set to p_A. Similarly, during the night we have probability p_B for B_{ij} activation. The result is a stochastic pattern of potentially

infectious interactions, in which the idling time between subsequent instances of activity follows a geometric distribution Geom(p_A) or Geom(p_B) for A_{ij} and B_{ij}, respectively. On average, we have A_{ij} links active

$$T_1 = 12p_A \tag{1}$$

hours, and B_{ij} active for

$$T_2 = 12p_B \tag{2}$$

hours, per daily cycle.

There are two exceptions to the above random activation rules:

- **Isolation**. In case node i is known to be infected, *i.e.* symptomatic (Sec. 1.3), then i's entire household $h(i)$ enters isolation. All nodes in $h(i)$ remain at home until the household is cleared to retain its activity. Under these conditions only B_{ij} is activated with probability p_B throughout the entire 24 hour cycle, and A_{ij} links remain idle. Consequently, when in isolation, in-house interactions become more extensive, as they have more potential instances of infection, then during periods of normal activity.
- **Collocation**. For consistency, if at a certain instance, both links (i, j) and (i, k) are simultaneity active, then the triad link (j, k) is also activated. Indeed, a concurrent collocation of i, j and i, k, implies, by transitivity, an inevitable collocation also of j, k. This alllows us to capture potential correlations in the temporal patterns of the interactions.

A summary of all temporal network parameters appears in Supplementary Table 1.

1.2 Mitigation

During mitigation, the quarantined households express only B_{ij} throughout the 24 hour cycle, with all their A_{ij} links rendered inactive. If a household member is defective, their A_{ij} links continue to activate as usual. Partitioning the population, as in AQ or HQ, for example, is done at household level - namely households are randomly split among the cohorts. In each realization, we instigate the mitigation at a time t_0 when the fraction of infections $I(t = t_0)$ exceeds a *significant* threshold. We set this threshold at

$$I(t = t_0) = \frac{\ln N}{N}, \tag{3}$$

namely the time point where the total infected population is of the order of $\ln N$.

Parameter	Description	Value
N	Population size	10^4
M	Number of households	4×10^3
$p(k)$	A_{ij} degree distribution	Erdős-Rényi or scale-free
$p(m)$	Household size distribution	Empirically obtained, Supp. Table 5
$\langle k \rangle$	Average A_{ij} degree	15
$\langle m \rangle$	Average household size	2.5
p_A	Probability of A_{ij} link activation	Varied
p_B	Probability of B_{ij} link activation	Varied
T_1	Mean daily infection time via A_{ij}	Eq. (1)
T_2	Mean daily infection time via B_{ij}	Eq. (2)
α	Fraction of in-house infections	Extracted from data/simulation
β	infection growth rate	Extracted from data/simulation

Supplementary Table 1: **The parameters governing the temporal network**. We list the relevant quantities underlying our temporal network framework. Parameters that are unknown are varied to capture the breadth of different epidemiological scenarios. For example $P(k)$ is set to be both bounded (main text) or scale-free (Sec. 2); p_A, p_B are varied in our simulations. Parameters α, β are not set, but rather extracted from the observed spread, as explained in Sec. 1.4.

1.3 Disease dynamics

We begin with a fully susceptible ($\mathbb{S}$) population, and introduce a small fraction of exposed ($\mathbb{E}$) individuals. The potential transitions that ensue are shown in Supplementary Figure 1, whose main transitions include:

- **Infection**. At any encounter between a susceptible node i and a pre-symptomatic or infected node j, i may become exposed. By *encounter* we relate to an instance Δt in which the i, j link in A_{ij} *or* B_{ij} is active. The probability of infection at each encounter depends on the nature of the link, set to p_1 for A_{ij} links and p_2 for B_{ij}. External interactions A_{ij}, between associates, are typically less physical than in-house interactions between *e.g.*, family members, hence, typically $p_2 > p_1$. In practice, however, we can incorporate these probabilities into the encounter probabilities themselves, p_A, p_B. Indeed, stating that i and j interact with probability p_A and then infect with probability p_1, is equivalent to setting their interaction probability to $p_A p_1$, and having infections occurring with 100% certainty. Hence, for simplicity we set $p_1 = p_2 = 1$, and encapsulate the infection probabilities within the parameters T_1, T_2 in (1) and (2).

- **Infection classification**. During the simulation of the spread we keep count of the type of each infection. Infections occurring via B_{ij} links add to the in-house infection count θ_{In}; infections occurring out of home, through A_{ij} contribute to θ_{Out}.

- **Infection cycle**. Once a node becomes exposed it begins to transition between states according to Supplementary Figure 1a. Exposed nodes have contracted the virus, but are not yet infectious. These nodes are randomly split between $\mathbb{E}_{\rm S}$ with probability $p_{\rm S}$ and $\mathbb{E}_{\rm AS}$ with probability $p_{\rm AS} = 1 - p_{\rm S}$. This decides whether these nodes are *pre-symptomatic*, and eventually *will* develop symptoms, or *asymptomatic*, reaching recovery $\mathbb{R}$ without even experiencing symptoms. The remaining disease cycle continues according to the illustration. For example, nodes in $\mathbb{E}_{\rm S}$ will later transition to one of the infected stated $\mathbb{I}_{\rm M}, \mathbb{I}_{\rm S}$ or $\mathbb{I}_{\rm C}$ with probabilities $p_{\rm M} = 0.55, p_{\rm S} = 0.1$ and $p_{\rm C} = 0.05$, respectively; the remaining 30% are accounted for in the $\mathbb{E}_{\rm AS}$ trajectory. Similarly, $\mathbb{I}_{\rm S}$ nodes, after some time enter the hospitalized state $\mathbb{H}$, after which the recover with probability $p_{\rm HR} = 0.85$, and decease with probability $p_{\rm HD} = 0.15$.

  Note that ventilated individuals $\mathbb{V}$ are, by definition, also hospitalized. However, in out implementation we consider these as two isolated groups, *i.e.* ventilated vs. hospitalized without ventilation. Therefore, at all times we have $\mathbb{S}(t) + \mathbb{E}(t) + \mathbb{I}_{\rm NS}(t) + \mathbb{I}(t) + \mathbb{R}(t) + \mathbb{V}(t) + \mathbb{H}(t) + \mathbb{D}(t) = N$, comprising the entire population. Presenting our results we used the normalized compartments $S(t) = \mathbb{S}(t)/N, E(t) = \mathbb{E}(t)/N, I_{\rm NS}(t) = \mathbb{I}_{\rm NS}(t)/N, \dots$, which satisfy

  $$S(t) + E(t) + I_{\rm NS}(t) + I(t) + R(t) + V(t) + H(t) + D(t) = 1. \qquad (4)$$

- **Transition times**. The amount of time a node remains at a state $\mathbb{X}$ (other than $\mathbb{S}$) is chosen at random according to probability density $P_{\mathbb{X}}(t)$. We identify specific processes for which variability in the transition time may impact the effectiveness of alternating quarantine (AQ). For example, the time from exposure to infectiousness, or the time for asymptomatic individuals to recover are crucial. Deviations from the mean in these transition times may interfere with AQ's disease cycle synchronization. For instance, if a node remains asymptomatic for, *e.g.*, 3 weeks, which is beyond the average time to recovery, its infectiousness may spillover between AQ's subsequent activity cycles, allowing it to resume activity while still infectious. Similarly, if the pre-symptomatic stage is extended significantly beyond the 5 day average, an infected individual in week 1 may not yet develop symptoms during their isolation at week 2, once again, reducing the efficiency of AQ in removing *invisible spreaders*. Therefore, for these highly relevant transitions we placed a special emphasis to avoid underestimating their potential time-scale heterogeneity. In particular we identified four relevant processes: $P_{\mathbb{E}_{\rm AS}}(t)$, the time until an exposed asymptomatic individual ($\mathbb{E}_{\rm AS}$) becomes infectious ($\mathbb{I}_{\rm AS}$); $P_{\mathbb{I}_{\rm AS}}$, the time until an asymptomatic infectious node recovers; $P_{\mathbb{E}_{\rm S}}(t)$, the time until an exposed pre-symptomatic individual ($\mathbb{E}_{\rm S}$) becomes infectious ($\mathbb{I}_{\rm PS}$) and $P_{\mathbb{I}_{\rm S}}(t)$, the time for an infectious pre-symptomatic ($\mathbb{I}_{\rm PS}$) to show symptoms ($\mathbb{I}_{\rm M}$, $\mathbb{I}_{\rm S}$ or $\mathbb{I}_{\rm C}$). For these four functions we used a Weibull distribution, as explained in Sec. 4.1. This distribution allows us to capture the potentially variable time-scales across the population, thus

testing AQ under realistically challenging conditions.

1.4 Evaluating α and β

Our model parameters control the spreading dynamics via the temporal probabilities p_A and p_B, and their subsequent T_1 and T_2 in (1) and (2), that govern the rate of infections in and out of home. Once these parameters are set, the simulating results of the unmitigated spread allow us to evaluate the infection growth rate β and the in-house transmission rate α:

- **In-house infection rate** α. During the simulation we keep count of the source of all infections. Infections occurring via A_{ij}, amounting to θ_{Out} are external, while those that transmit along B_{ij} links, θ_{In}, are internal. The parameter α captures the percentage of transmissions that occurred in-house as

$$\alpha = \frac{\theta_{\text{In}}}{\theta_{\text{In}} + \theta_{\text{Out}}}. \quad (5)$$

- **The infection growth rate** β. To evaluate β we observe the overall infected population $I(t)$ vs t at the early stages of the spread, and fit it to an exponential of the form

$$I(t) \sim e^{\beta t}. \quad (6)$$

  Obtaining the slope of the resulting growth on semi-logarithmic axes we extract β from the simulation results. Note that β depends on the *slope*, not on the pre-factor, hence it is insensitive to the size of the initial outbreak, or to the fraction of cases detected via testing, providing a fair comparison between different realizations or empirical datasets.

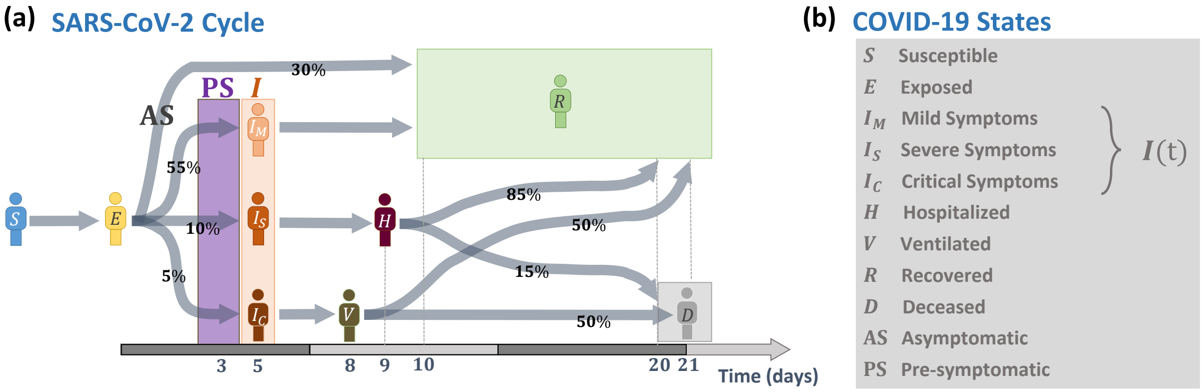

Supplementary Figure 1: The infection cycle of SARS-CoV-2. Extracted from Fig. 1 of the main text.

A crucial point is to select the range in t from which to extract the slope. Indeed, for very small t, due to the stochastic nature of our simulations, $\mathbb{I}(t)$ is still small, and still discrete. In this limit, the observed results are subject to high levels of noise and may not yet exhibit a clear exponential behavior. On the other hand if t is too large, we approach the peak of $I(t)$, where the exponential approximation fails again, this time due to the accumulation of herd immunity. Therefore, to be consistent across all our simulations we evaluated β from the time window

$$\frac{t^\star}{4} \le t \le \frac{t^\star}{2} \tag{7}$$

where $t^\star = \arg\max I(t)$ is the time of peak infection. Evaluating β from empirical data is explained in Sec. 4.

Note, that α and β are *not* the model parameters. Rather they emerge from the stochastic simulation results, after setting the model parameters p_A and p_B. Therefore, we do not have direct control over these parameters, as seen in, *e.g.*, Fig. 4 of the main text, where α, β were only approximately equal across the different panels. Roughly speaking, we can link these parameters to each other. A large p_A, p_B, for example enhances transmission, and hence increases β. The parameter α, on the other hand, grows as p_B is increased and p_A is decreased, capturing a state in which in-house transmissions are more prevalent than external ones.

1.5 Evaluating R_0

Our disease cycle is elaborate and includes many different paths. It is, therefore difficult to accurately calculate the reproduction number R_0. Instead we use an approximate approach, based on an SIS-like rationale. Consider the SIS model [2] and its two parameters: ω, the recovery rate, and μ, the infection rate. In this model we have

$$R_0 = \frac{\mu}{\omega}, \tag{8}$$

capturing the number of transmissions that an average individual generates (μ) during their period of infectiousness, which typically lasts for $\tau = 1/\omega$ days. The exponential growth rate in the SIS model at the early stages of the outbreak is given by $I(t) \sim e^{\beta t}$ as in (6) with

$$\beta = \mu - \omega. \tag{9}$$

To adapt (8) to our more complex disease cycle we seek the relevant period of infectiousness τ that arises from the cycle of Supplementary Figure 1. To evaluate τ we track the path of the average individual: with probability 0.3 this individual is asymptomatic, in

which case they will remain infectious until their recovery for an average of 7 days; an additional ~ 0.3-fraction infect at home (θ_{In}), here over an average period of roughly 8 days. The remaining 0.4-fraction of infected individuals cease to transmit the virus once they enter isolation, on average 2 days posterior to the onset of the pre-symptomatic phase. Together we approximate the period of infectiousness as

$$\tau = 0.3 + 0.3 \times 8 + 0.4 \times 2 = 5.3 \ \text{days}, \tag{10}$$

allowing us to evaluate the effective recovery rate by

$$\omega_{\mathrm{eff}} = \frac{1}{\tau} = 0.19 \ \text{days}^{-1}. \tag{11}$$

We can now substitute ω in (8) and (9) with ω_{eff} to write

$$\begin{cases} R_0 & = & \dfrac{\mu}{\omega_{\mathrm{eff}}} \\ \\ \beta & = & \mu - \omega_{\mathrm{eff}} \end{cases}, \tag{12}$$

which, setting $\omega_{\mathrm{eff}} = 0.19$ and $\beta = 0.26$ provides $\mu = 0.45$ and

$$R_0 \approx 2.4. \tag{13}$$

Supplementary Note 2

Results obtained under a scale-free A_{ij}

Our strategy in testing AQ is to examine it systematically under varying relevant scenarios. Specifically, for unknown parameters, such as α and β, we simulated an array of different setting, scanning the space of potential α, β values (*e.g.*, Fig. 4 of main text). Other unknown factors relate to the structural characteristics of the external network A_{ij}. Most importantly, in the context of epidemic spreading - its degree distribution, which has been shown to significantly impact the patterns of spread [2]. To eliminate this potentially confounding factor we now re-examine AQ, repeating our simulations, this time extracting A_{ij} from the scale-free network ensemble ($P(k) \sim k^{-3}, N = 10^4, \langle k \rangle = 15$). We find, in Figs. 2 and 3 that AQ continues to provide the optimal mitigation also under these conditions.

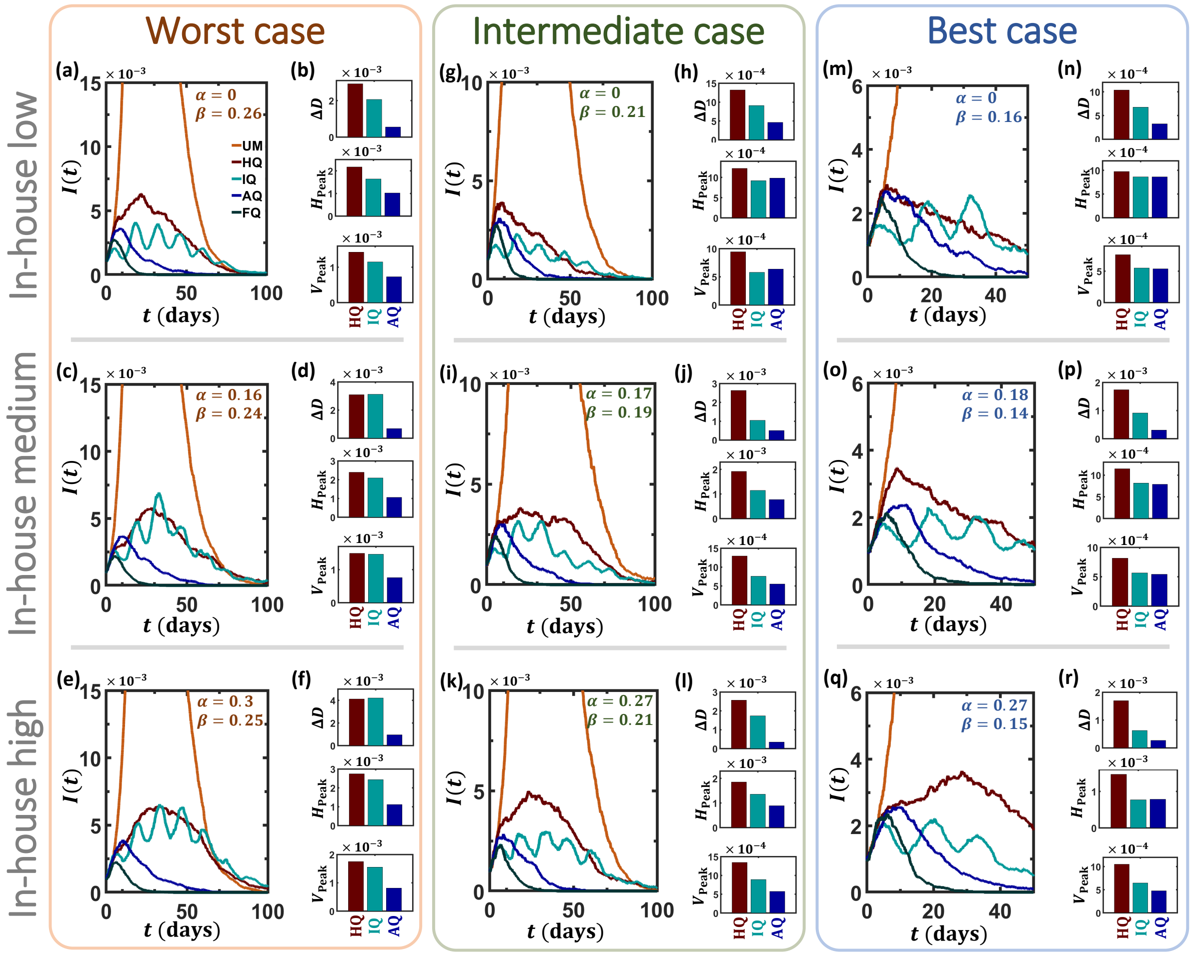

Supplementary Figure 2: The impact of alternating quarantine for a scale-free A_{ij}. Reconstructing Fig. 4 of the main text, this time using a scale-free external network.

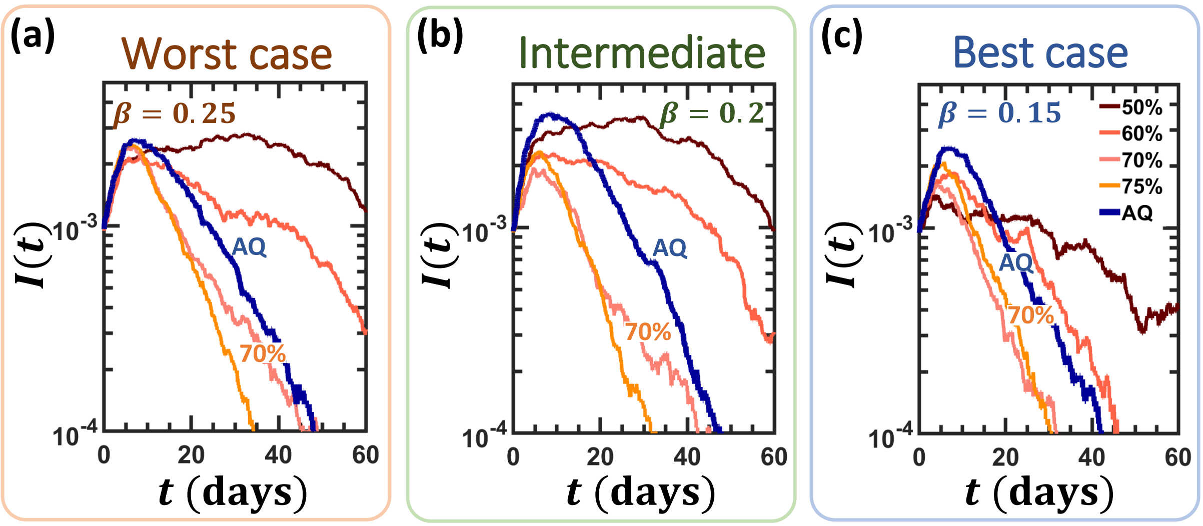

Supplementary Figure 3: **Alternating quarantine vs. population-wide quarantine on a scale-free** A_{ij}**.** Reconstructing Fig. 7 of the main text, this time using a scale-free external network.

Supplementary Note 3

Synergistic measures

3.1 Alternating quarantine under selective isolation

In the main take we used a *typical* disease cycle, capturing the average individual's response to SARS-CoV-2. We now consider two parallel cycles, one for healthy individuals and the other for the vulnerable, such as people with background diseases or the elderly. The two cycles differ mainly in their transition probabilities. For example, while only 4% of the healthy individuals develop critical symptoms ($\mathbb{I}_C$), among the vulnerable population the number is set to 10%. The complete disease cycle for the Typical, Healthy and Vulnerable population [3–5] appears in Supplementary Table 2.

To track the spread in the presence of healthy/vulnerable populations we repeated the simulation described in Sec. 1, this time splitting the population into 80% healthy and 20% vulnerable nodes [6]. We track three indicators that help us assess the performance of all strategies (Supplementary Figure 4): Hospitalization rate $H(t)$, ventilation rate $V(t)$ and mortality $D(t)$. As expected, AQ (Supplementary Figure 4)a,d,g, light blue) continues to outperform IQ (light turquoise) and HQ (light red) also under this variable disease cycle.

Next, we added an additional component of selective isolation, in which the vulnerable nodes (20%) remain under constant quarantine. For example, in AQ this implies that the weekly alternations are limited only to the healthy 80%. Under these conditions the vulnerable individuals cannot be infected via external links A_{ij}. They can still, however, experience secondary infection through B_{ij}, in case one of their healthy cohabitants contracted the virus. As expected, such selective isolation enhances the performance of all the strategies, lowering hospitalization, ventilation and mortality (Supplementary Figure 4b,e,h). This improvement, we emphasize, is not unique to AQ, making it clear that selective isolation is a desirable component within any mitigation strategy. In Supplementary Figure 4c,f,i we present our three performance measures, H_{Peak}, V_{Peak} and ΔD with (dark) and without (light) selective isolation, further indicating the importance of protecting the vulnerable population.

3.2 Alternating quarantine with population-wide testing

Thanks to the synchronization with the disease cycle, each weekly quarantine filters out a fraction of the infected individuals. It is therefore natural to reinforce this filtering with systematic testing of the quarantined cohort before they resume activity. If an individual is detected positive, their entire household must remain in isolation until all members are cleared. To examine this effect we added a component of random testing to both AQ

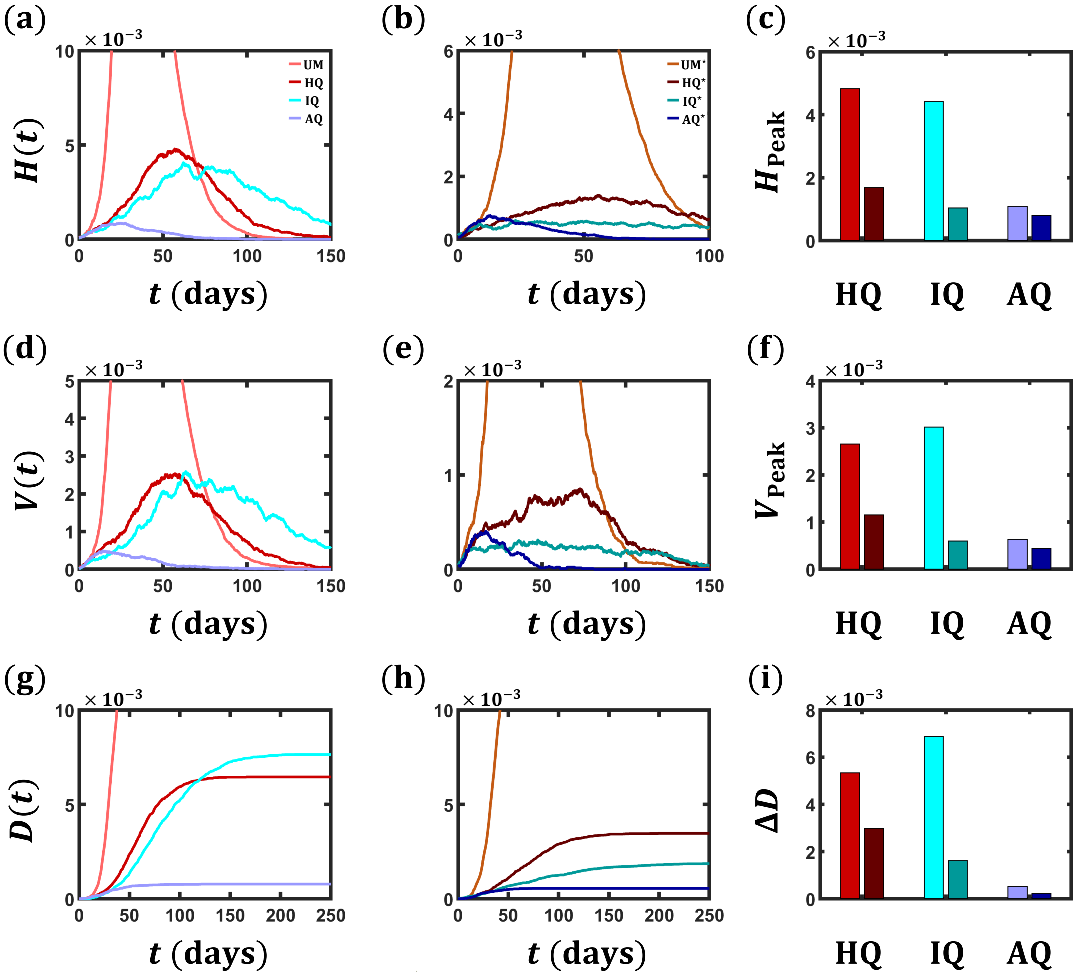

Supplementary Figure 4: **The impact of selective isolation on the different mitigation approaches**. (a) The fraction of hospitalized individuals $H(t)$ vs. t under no mitigation (light orange, UM), intermittent quarantine (IQ, light turquoise), half quarantine (HQ, light red) and alternating quarantine (AQ, light blue). (b) Similar results (dark colors), this time with selectively isolating the vulnerable population. (c) Peak hospitalization under all strategies with (light) and without (dark) selective isolation. (d) - (f) Ventilated population $V(t)$ vs. t with/without selective isolation. (g) - (i) Mortality $D(t)$ and the residual mortality ΔD.

**Probability**	**Typical**	**Healthy**	**Vulnerable**
p_{AS}	0.3	0.32	0.25
p_M	0.55	0.56	0.45
p_S	0.1	0.08	0.2
p_C	0.05	0.04	0.1
p_{HR}	0.85	0.86	0.79
p_{HD}	0.15	0.14	0.21
p_{VR}	0.5	0.5	0.5
p_{VD}	0.5	0.5	0.5

Supplementary Table 2: **Transition probabilities between COVID-19 states.** We constructed three disease cycles - the Typical cycle, used in the main text, the Healthy cycle, capturing the impact of the disease on healthy individuals, and the Vulnerable cycle, adapted to individuals of age or ones with pre-existing conditions.

and IQ. Given the limited resources, we assume a testing capacity of a χ-fraction of the population per week. As expected, we find that the greater is χ the more effective is our mitigation (Supplementary Figure 5a,b).

The crucial point, however, is that AQ's breakdown of the population into separated cohorts provides an intrinsic advantage. Indeed, testing is most effective when conducted on the quarantined cohort, whose state is *frozen* during the week. One can then spread the testing across the entire week, and detect infected individuals before they return to activity. Hence, the fact that one only needs to focus on half of the population at a time, enhances the effectiveness of such a testing policy. To understand this, consider the case where $\chi \approx 0.5$, namely we have the capacity to test 50% of the population within a single week. Under these conditions, thanks to AQ's partitioning, one can simply invest *all* tests in the inactive cohort, then resume activity in week 2 with a guaranteed clean workforce.

To examine this advantage we focus specifically on the case where $\chi = 0.5$. We apply the tests selectively to the quarantined cohort in each shift, which, indeed, constitutes roughly half of the population (minor discrepancies arise due to statistical variations, and uneven household sizes). Within a one week cycle we arrive at an almost 100% uninfected active workforce, after which the only bottleneck for the decay of $I(t)$ is the residual in-house infections. In that sense, after approximately $1-2$ weeks, AQ becomes as effective as FQ. Indeed, Supplementary Figure 5c shows that AQ (blue) exhibits the same rate of $I(t)$ decay as FQ (grey), albeit at a 10 day delay, precisely the predicted $1-2$ weeks. Hence, extensive testing provides a crucial complement to AQ, potentially achieving FQ mitigation efficiency, without crippling socioeconomic activity.

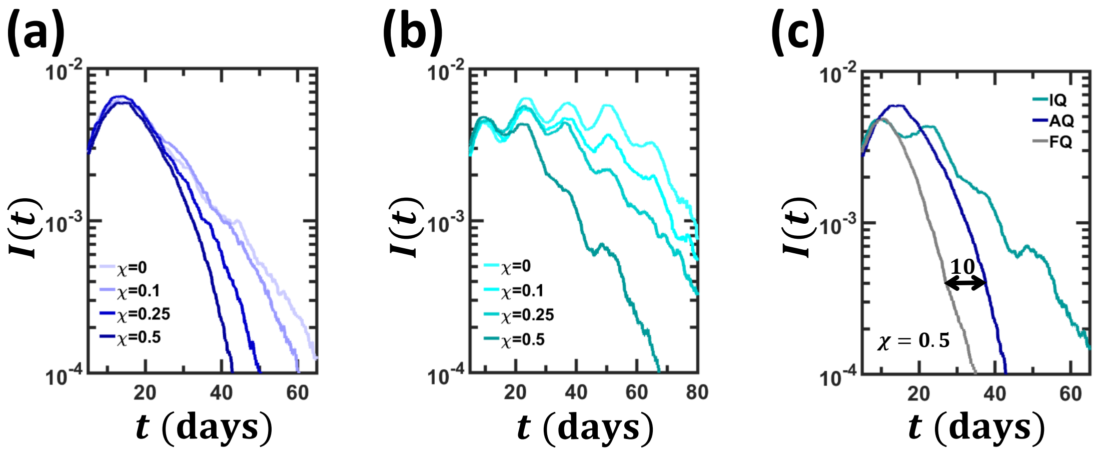

Supplementary Figure 5: **Testing the population before resuming activity**. (a) We combine AQ with testing at a weekly capacity of χ. Directing all tests towards the quarantined cohort at each week, we prohibit individuals who tested positive (and their households) from resuming activity. Unsurprisingly, as χ increases (darker) we observe an enhanced mitigation, thanks to the systematic pruning of infected individuals from the active cohort. (b) Testing also improved the performance of Intermittent quarantines (IQ). As opposed to AQ, in IQ, as the entire population transitions from quarantine to activity in unison, the testing cannot be selectively directed to the quarantined cohort, but rather spread evenly across the entire population. (c) In the limit where $\chi \to 0.5$, a capacity to screen 50% of the population within one week, AQ becomes extremely efficient, thanks to its natural partitioning of the population. The entire quarantined cohort can be tested, and within $1-2$ weeks AQ has almost no out of home infections. Indeed, we observe that AQ follows a similar decay as the Full quarantine (FQ, grey), albeit with a 10 day delay, capturing roughly $1-2$ testing cycles. In contrast, IQ, lacking such partitioning of the population, exhibits a more minor benefit under the same testing capacity. Simulations represent an average over 20 stochastic realizations. The in-house infection rate was set to the intermediate level $\alpha \approx 0.15$.

Supplementary Note 4

Data analysis and parameter selection

4.1 Constructing the distributions $P_{\mathbb{X}}(t)$

Most of the parameters described in Section 1 were chosen based on observed values of the characteristic SARS-CoV-2 infection cycle. For the density functions $P_{\mathbb{X}}(t)$, we have used a Weibull or a Geometric distribution, the former - inspired both by other infections of the Corona variety [7], as well as recent inidcations pretainig to SARS-CoV-2 [8–10]. The Weibull distribution allows for potentially high variability across the population, providing a *challenging* testing ground for AQ.

To estimate the parameters of the Weibull distributions we collected data on the average T_{Av} and median T_{Med} of the relevant transition times [7, 8]. This allowed us to infer the Weibull parameters λ and k via

$$T_{\text{Av}} = \lambda\Gamma(1 + 1/k);$$
$$T_{\text{Med}} = \lambda(\ln 2)^{1/k}.$$

As median values were available only for $P_{\mathbb{I}_{\text{AS}}}$ and $P_{\mathbb{I}_{\text{PS}}}$, we first calculated the parameter k for these transitions, obtaining, for both $k = 1.47$. This is not surprising as k, the *shape* parameter, controls the type of the Weibull distribution, which is expected to be similar for processes driven by similar mechanisms. This is as opposed to λ, the *location* parameter, which is not intrinsic to the shape of the distribution, but rather shifts *right* or *left* as the mean is changed. Hence, it is expected that k is uniform for the different transition-time distributions, while λ may change according to their mean. With this in mind, we estimated $k = 1.47$ for the other two distributions, $P_{\mathbb{E}_{\text{AS}}}$ and $P_{\mathbb{E}_{\text{S}}}$, where the median was inaccessible from data. See Supplementary Table 3 for the different values of mean, and median we have used, and the inferred λ and k.

4.2 Estimating the infection growth β

As defined above, the parameter β represents the exponential growth rate of the known infectious nodes $I(t) := I_M(t)+I_S(t)+I_C(t)$. This parameter is difficult to predict directly from the knwon disease time-scales, especially as the infection rate is hidden, hence we must infer it from observation. Moreover, as the disease progresses, precautions like social distancing and wearing masks affect both the rate of interaction and the probability of infection, leading β to change over time. Therefore, to asses β for the unmitigated spread, we have focus on the period *before* such measures were taken.

We collected data on the number of confirmed cases in 12 countries. These countries

Duration	Distribution	Observations		Parameters	
		Mean	Median	λ	k
$P_{\mathbb{E}_{AS}}(t)$	Weibull	4	3.44*	4.42	1.47*
$P_{\mathbb{I}_{AS}}(t)$	Weibull	10	8.6	11.04	1.47
$P_{\mathbb{E}_S}(t)$	Weibull	2	1.72*	2.21	1.47*
$P_{\mathbb{I}_{PS}}(t)$	Weibull	5	4.3	5.52	1.47
		Mean		p	
$P_{\mathbb{I}_M}(t)$	Geometric	5		0.2	
$P_{\mathbb{I}_S}(t)$	Geometric	4		0.25	
$P_{\mathbb{I}_C}(t)$	Geometric	3		0.333	
$P_{\mathbb{I}_H}(t)$	Geometric	11		0.091	
$P_{\mathbb{I}_V}(t)$	Geometric	13		0.077	

Supplementary Table 3: **Estimating the distribution parameters**. With data on the mean and median transition times, we reconstructed the distributions $P_{\mathbb{X}}(t)$. For the first four transitions we used a Weibull distribution, since the potentially high variability is key for testing AQ. The remaining distributions were taken to be Geometric, since only the mean matters for these transitions. Asterisked median values are reconstructed.

have been selected for their prominent number of casesm and to obtain a balanced representation between southern and northern hemisphere destinations. The data set was compiled by and obtained from the Johns Hopkins University Center for Systems Science and Engineering (JHU CSSE) on April 11th 2020 and is available online here: *https://data.humdata.org/dataset/novel-coronavirus-2019-ncov-cases* [11].

To capture the relevant time-window for the exponential growth approximation we used data-points starting 5 days before lock-down and ending 3 days after it. Indeed, earlier than this point, cases may be underestimated by a yet unprepared system, and beyond this window, the lock-down may begin affecting the observed slope. As clearly seen in Fig. 3 of the main text, within this time-window the spread $I(t)$ can be well-approximated by an exponential growth of the form (6). To extract the slope we used linear regression on $\ln I(t)$, yielding the estimator $\hat{\beta}$ for the growth rate in each country, as detailed in Supplementary Table 4 and in Supplementary Figure 6. We find that estimators are narrowly distributed around an average of $\beta = 0.26$, the value we used as our *default*, *i.e.* unmitigated spreading parameter.

4.3 Estimating the household size distribution $P(m)$

We used a United Nations database [12] to collect data on the distribution of household sizes across different countries. The data, summarized in Supplementary Table 5, was compiled by and obtained from the United Nations, Department of Economic and Social Affairs, Population division.

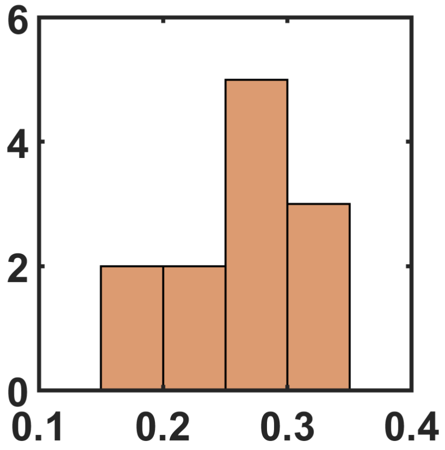

Supplementary Figure 6: **The variability of infection rates across countries**. Histogram of the estimator $\hat{\beta}$ values by countries.

Country	Population	First case	Lock-down	$\hat{\beta}$
Italy	60	10	38	0.32
USA	328	3	61	0.3
Spain	47	19	43	0.34
Israel	9	36	54	0.19
Germany	83	7	52	0.26
Norway	5.4	38	49	0.32
Colombia	52	49	56	0.28
Argentina	45	45	50	0.22
Netherlands	17	39	54	0.21
N. S. Wales	8	5	52	0.18
Austria	9	35	45	0.3
UK	56	10	46	0.29

Supplementary Table 4: **Estimating** β **per country**. Population is given in millions. First case and Lock-down are given in days relative to 22/1. The parameter $\hat{\beta}$ represents the estimation for β, as extracted from the relevant country data. See Supplementary Figure 6 for a histogram of $\hat{\beta}$.

**Country**	1	2-3	4-5	6	Average
Italy	0.31	0.47	0.21	0.01	2.4
Germany	0.39	0.47	0.13	0.01	2.05
USA	0.28	0.49	0.19	0.04	2.5
Israel	0.21	0.4	0.28	0.11	3.14
Spain	0.19	0.53	0.26	0.02	2.69
Norway	0.4	0.41	0.18	0.01	2.22
Model	0.3	0.46	0.2	0.04	2.6

Supplementary Table 5: **Household size distribution** $P(m)$ **per country**. For each country we show the fraction of households with 1, $2-3$, $4-5$ or 6 cohabitants, as obtained from the UN database [12]. We also show the average household size in each country.

References

[1] M.E.J. Newman. *Networks - an introduction.* Oxford University Press, New York, 2010.

[2] R. Pastor-Satorras, C. Castellano, P. Van Mieghem and A. Vespignani. Epidemic processes in complex networks. *Rev. Mod. Phys.*, 87:925–958, 2015.

[3] S. Mallapaty. The coronavirus is most deadly if you are older and male - new data reveal the risks. *Nature*, page 16, 2020.

[4] M. Bonafè *et al.* Inflamm-aging: why older men are the most susceptible to sars-cov-2 complicated outcomes. *Cytokine Growth Factor Review*, page 33, 2020.

[5] Y. Xie *et al.* Epidemiologic, clinical, and laboratory findings of the covid-19 in the current pandemic: systematic review and meta-analysis. *BMC Infect Dis.*, page 640, 2020.

[6] United Nations report. World population ageing 2019.

[7] Yinon M Bar-On, Avi Flamholz, Rob Phillips, and Ron Milo. Sars-cov-2 (covid-19) by the numbers. *eLife*, 9, 2020.

[8] Natalie M Linton, Tetsuro Kobayashi, Yichi Yang, Katsuma Hayashi, Andrei R Akhmetzhanov, Sung-mok Jung, Baoyin Yuan, Ryo Kinoshita, and Hiroshi Nishiura. Incubation period and other epidemiological characteristics of 2019 novel coronavirus infections with right truncation: a statistical analysis of publicly available case data. *Journal of clinical medicine*, 9, 2020.

[9] S.A. Lauer *et al.* The incubation period of coronavirus disease 2019 (COVID-19) from publicly reported confirmed cases: estimation and application. *Ann Intern Med.*, 172:577–582, 2020.

[10] Jantien A Backer, Don Klinkenberg, and Jacco Wallinga. Incubation period of 2019 novel coronavirus (2019-ncov) infections among travellers from wuhan, china, 20–28 january 2020. *Eurosurveillance*, 25, 2020.

[11] E. Dong, H. Du and L. Gardner. An interactive web-based dashboard to track COVID-19 in real time. *The Lancet Infectios Diseases*, 2020.

[12] United Nations Department of Economic and Social Affairs. Household size and composition 2019. https://population.un.org/Household/index.html/countries/840.
